# Impact Behavior of Three Notched All-Ceramic Restorations after Soaking in Artificial Saliva

**DOI:** 10.3390/ma8074479

**Published:** 2015-07-20

**Authors:** Min Yan, Chung-Kai Wei, Yuh-Yih Lin, Suh-Woan Hu, Shinn-Jyh Ding

**Affiliations:** 1 Institute of Oral Science, Chung Shan Medical University, Taichung City 402, Taiwan; E-Mails: yan@csmu.edu.tw (M.Y.); suhwoan@csmu.edu.tw (S.-W.H.); 2 Department of Dentistry, Chung Shan Medical University Hospital, Taichung City 402, Taiwan; E-Mails: tiyeg2@gmail.com (C.-K.W.); yuhyih@csmu.edu.tw (Y.-Y.L.); 3 School of Dentistry, Chung Shan Medical University, Taichung City 402, Taiwan

**Keywords:** all-ceramic restorations, impact energy, leucite-reinforced ceramic, lithium disilicate ceramic, zirconia, saliva

## Abstract

Biomechanics play a critical role in influencing the clinical applications of all-ceramic dental restorations. The restorative biomaterials have to demonstrate mechanical durability in the oral environment because they are always exposed to a variety of oral environments. This study was designed to evaluate the effect of soaking time, notch and saliva pH values on the impact energy of three commonly used all-ceramic materials for CAD/CAM. The leucite-reinforced glass ceramic (ProCAD), lithium disilicate glass ceramic (IPS e.max CAD) and zirconia-based ceramic materials (IPS e.max ZirCAD) were used. The experimental results indicated that the impact energy of ProCAD decreased with an increase in soaking time, but not for IPS e.max CAD and IPS e.max ZirCAD. The impact energy of the zirconia system was higher than leucite-reinforced and lithium disilicate-based ceramic systems. When subjected to preformed 0.5 mm U-shape notch on the bar specimen of 3 mm thick, the impact energy of the all-ceramic restorations revealed a markedly reduction of about 80%–90%, almost irrespective of dental compositions, which indicated the effect of flaw to a great degree. No statistically significant influence (*p* > 0.05) of pH values (4, 7 and 9) on impact energy was found for each group. It is concluded that the no matter which all-ceramic materials were used, it was appreciably sensitive to the presence of notches. The ceramic composition and microstructure have been shown to affect mechanical durability.

## 1. Introduction

All-ceramic restorations have been used in dentistry due to their unique properties, such as a very translucent and natural appearance, high resistance to wear and distortion, chemical stability, thermal stability and excellent biocompatibility [1,2,3]. The dental restorations have been used for numerous clinical applications, including inlays, crowns, veneers, three- and four-unit bridges, and implants [2,3,4,5]. Especially, all-ceramic crowns could avoid either gingival discoloration around the margins crown or allergic responses compared to porcelain-fused-to-metal (PFM) crowns [6]. Although all-ceramic materials have a high strength and fracture toughness, the dental clinical researches have reported that the main reason for failure originates from the restoration fracture, such as veneering porcelain, ceramic coping, and the connector for fixed partial denture prosthesis (FPDPs) [7,8,9,10]. A systematic review for currently used ceramic materials has indicated that typical fracture rates for all-ceramic restorations are still great ranging from 0 to 12% after two to five years in service, and 3% to 16% after 5–14 years in service [7]. An inherently brittle ceramic has innate microscopic flaws on the surface and/or in the interior of the material, which formed during fabrication and treatments [11,12]. As a result, the flaws may be the predominant cause for reduction of mechanical strength and lifetime. Therefore, the effect of the existed damage or flaws on mechanical properties is worth evaluating from the viewpoint of clinical investigation. 

Flexural strength, fatigue behavior and fracture toughness are generally considered meaningful methods to assess the mechanical performance of materials [13,14]. On the other hand, when they are susceptible to the sudden force, an important property of all-ceramic restorations, the ability to resist high impact loading, needs to be concerned. In impact test, the samples are fixed at both ends to a metal holder, which is different from the mode of flexural strength tests. The measurement of the energy absorbed should be valuable in evaluating all-ceramic materials when the instantaneous impact fracture occurs. Aboushelib *et al*. [15] used a modified Charpy’s impact machine to investigate impact strength of two layered all-ceramic restorative systems. Nevertheless, little is known about the impact energy of all-ceramic dental materials to ascertain the feasibility of their long-term clinical applications. 

Because all-ceramic materials are always exposed to a variety of oral environments [16], it is indispensable for the examination of the mechanical stability *in vitro* influencing their clinical performance. Aforementioned, the mechanical properties of all-ceramic restorations depend noticeably on the presence of flaws and micro-cracks on the surface, which act as stress concentrators upon being loaded. The purpose of this study was to evaluate the impact energy of three preformed notched all-ceramic materials (ProCAD, IPS e.max CAD and IPS e.max ZirCAD) commonly used for CAD/CAM (computer-aided design and computer-aided manufacturing), including leucite-reinforced, lithium disilicate-based, and zirconia-based ceramics. To simulate the oral environment, the effect of soaking time in artificial saliva (pH 7) on the impact energy of all-ceramic materials was also evaluated. In addition, saliva of pH 4 and 9 were selected to simulate clinical conditions that would be considered extreme. The research hypothesis for this study was that notch and soaking time had effects on impact energy of the three all-ceramic materials.

## 2. Experimental Section

### 2.1. Materials

Three commercially all-ceramic systems for CAD/CAM were used in this study (Table 1). ProCAD (Ivoclar-Vivadent, Schaan, Liechtenstein) was a leucite reinforced ceramic. IPS e.max CAD and IPS e.max ZirCAD consisted mainly of lithium disilicate and zirconia phases, respectively, and were also purchased from Ivoclar-Vivadent. 

**Table 1:** Characteristics and applications of three all-ceramic dental restorations used in this study.

Brand	Lot No.	Crystal Phase	Sintering/Crystallization Temperature (°C)	Application
ProCAD	G17661	Leucite	625	inlay, onlay, single crown, veneer
IPS e.max CAD	J07273	lithium disilicate	850	inlay, onlay, veneer, single crown, bridge
IPS e.max ZirCAD	J20390	Zirconia	1500	inlay, onlay, single crown, three unit bridge

### 2.2. Sample Preparation

The bar-shaped specimens were fabricated using a low-speed diamond saw (Isomet, Buehler, Lake Bluff, IL, USA). Each specimen surfaces were polished with 280-grit SiC abrasive paper. The final dimensions of the specimens were 15 mm length × 4 mm width × 3 mm thickness. After that, the specimens were sintered according to the manufacturer’s suggestions.

**Figure 1:**
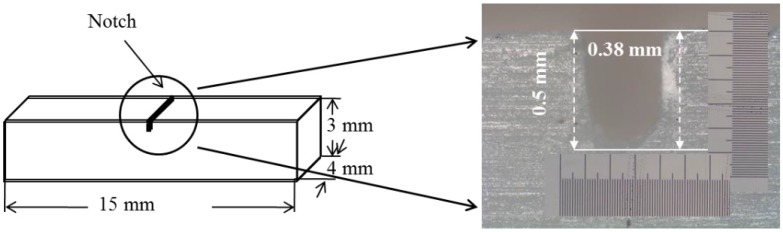
Shape and dimensions of the all-ceramic specimen, indicating the notch structure.

Aforementioned, a systemic evaluation of the notch effect on impact behavior of three all-ceramic materials was performed. The U-notch with 0.5 mm depth and 0.38 mm width (Figure 1) was made on the specimen surface toward the thickness direction at the center position using a low-speed diamond saw (Isomet, Buehler) with water. The introduction of about 0.5 mm depth was referenced to the literature [17,18,19], which indicated the ratio (0.2) of notch depth to sample thickness. The 0.38 mm size was the thickness of the saw blade.

The specimens were individually immersed in the artificial saliva [20] at 37 ± 0.2 °C for 30 and 90 days. The hydrochloric acid or sodium hydroxide was used to adjust the pH value to 4, 7 and 9 monitored by a pH meter (SP-701, Suntex Instruments, Taipei, Taiwan). After soaking, the specimens were removed from the vials to evaluate the impact energy and morphology.

### 2.3. Impact Test

To simulate crack-induced fractures, Charpy impact test was used to measure the impact energy absorbed by the materials [15,18,21]. A home-made pendulum impact tester with a loading of 103 g and an arm length of 122 mm was designed according to ISO standard 197 [19] and 13802 [22]. The specimens were horizontally positioned with a span distance of 10.5 mm between the two fixed supports. After the test specimen was placed on the sample base, the pendulum hammer was released from its rest at a given height to hit the specimen. The impact angle of the test specimen was monitored by protractor. The impact energy of each specimen was calculated by the following formula:

∆*E* = *WR* (cosβ-cosα) (1)

where ∆*E* is absorbed energy by the specimen during fracture (J); *W* is a load of the pendulum (Kg); *R* is the distance (m) between starting point and striking point; β is impact angle (°) and α is the starting angle (°). The tests were carried out at room temperature of 23.0 °C and 70% relative humidity. Five samples were tested for each condition.

### 2.4. Surface Morphology

After soaking in pH 7 artificial saliva for 90 days, the surface morphology of the specimens were coated with gold using a JFC-1600 (JEOL, Tokyo, Japan) coater and examined by field-emission scanning electron micrographs (JEOL JSM-7401F, Tokyo, Japan) operating in the lower secondary electron image mode (LEI) at 3 kV accelerating voltage. 

### 2.5. Statistical Analysis

The non-parametric Kruskal-Wallis test was used to compare median impact energy among groups with different soaking time. When the test result was significant, the Dunn procedure was applied for *post hoc* comparisons of specific groups. The Kruskal-Wallis test was done for each material with and without notch, respectively. Data analysis was performed using the JMP version 11 software (SAS Institute, Inc., Cary, NC, USA). A *p*-value of less than 0.05 was considered significant.

## 3. Results

### 3.1. Surface Morphology

**Figure 2:**
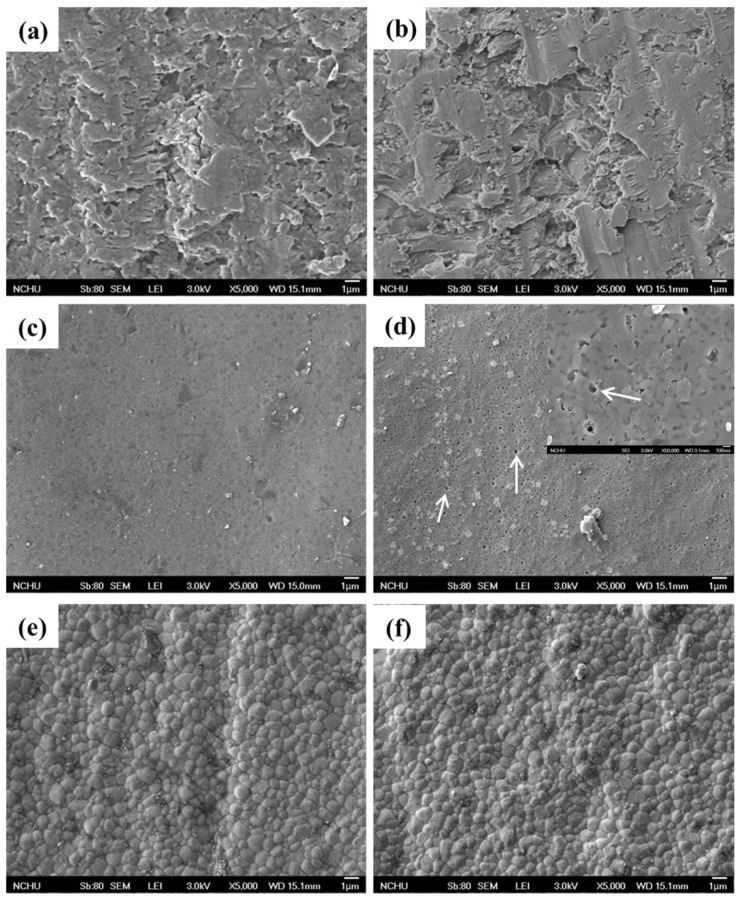
Surface micrographs of ProCAD (**a**,**b**), IPS e.max CAD (**c**,**d**) and IPS e.max ZirCAD (**e**,**f**) before (**a**,**c**,**e**) and after (**b**,**d**,**f**) soaking in pH 7 artificial saliva for 90 days. The arrows indicate the pitting pores. Magnification: 5 kx. The insert in (**d**) is a magnified picture with a magnification of 50 kx.

Figure 2 shows the surface morphologies of the three different all-ceramic materials before and after soaking in pH 7 artificial saliva for 90 days. To be reasonable here was to see the different surface morphologies of the materials before soaking, which depended on the materials characteristics. After soaking for 90 days, it seems that the ProCAD specimen exhibited the loss of surface structure (Figure 2b) compared to the respective control with a rough structure (Figure 2a). As for IPS e.max CAD, the formation of soaking-induced pitting pores (indicated by arrows) with diameter of about 0.2 μm (Figure 2d) was found on the as-prepared smooth surface (Figure 2c). The insert in Figure 2d shows a magnified picture to highlight the presence of the pitting pores. In contrast to the other two all-ceramic materials, IPS e.max ZirCAD exhibited the fine-grained structure with an average grain size of approximately 500 nm without a remarkable change before (Figure 2e) and after 90-day soaking (Figure 2f). More importantly, minor differences in structure were not found.

### 3.2. Soaking and Notch Effects

**Figure 3:**
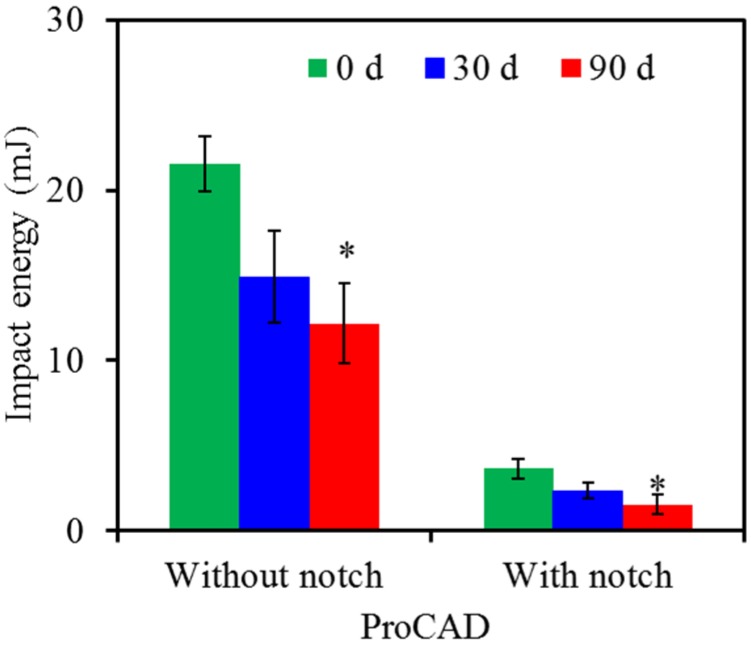
Impact energy of ProCAD without and with notch before and after soaking in pH 7 artificial saliva. Asterisk statistically significant difference (*p* < 0.05) from the control group without soaking.

**Figure 4:**
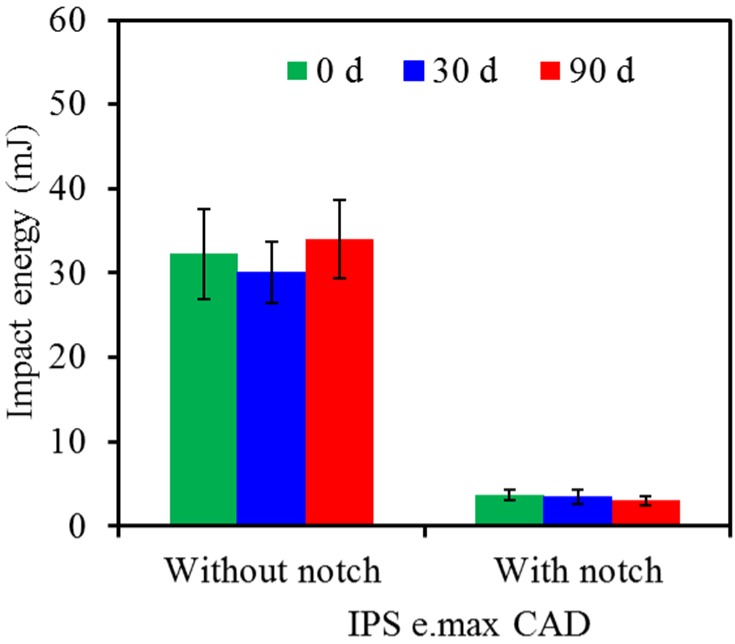
Impact energy of IPS e.max CAD without and with notch before and after soaking in pH 7 artificial saliva.

**Figure 5:**
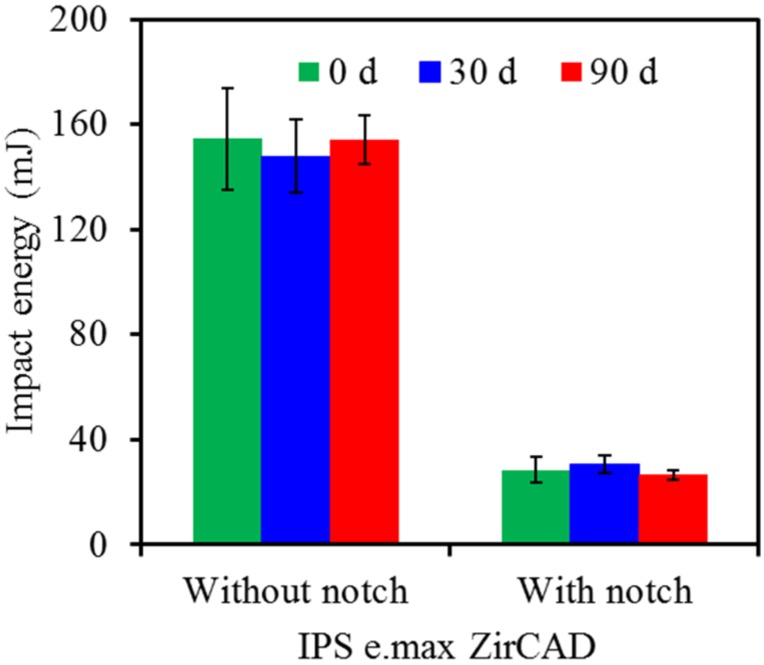
Impact energy of IPS e.max ZirCAD without and with notch before and after soaking in pH 7 artificial saliva.

Figure 3, Figure 4 and Figure 5 show impact energy values of three all-ceramic materials without and with notch before and after soaking in artificial saliva of pH 7 for 30 and 90 days. It can be clearly seen that the values of all all-ceramic restorations decreased significantly (*p* < 0.05) with the introduction of notch compared with those of the respective groups without the notch. Concerning ProCAD without the notch, the impact energy declined from the original value of 22 mJ to 15 and 12 mJ after 30-day and 90-day soaking, respectively (Figure 3). Similarly, after soaking for 30 and 90 days, the impact energy values of notched specimens became markedly 2.4 and 1.6 mJ, lower than the respective energy value at day 0 (3.7 mJ).

In the case of IPS e.max CAD, the values of the control without notch at 0, 30- and 90-day soaking were 32, 30 and 34 mJ, respectively (Figure 4), while the notched specimens decreased to the values of 3.7, 3.5 and 3.0 mJ. The effect of soaking time periods on the variations in the impact energy demonstrated no significant differences (*p* > 0.05) for either control specimens or notched specimens. We also found this to be the case for IPS e.max ZirCAD (Figure 5), although the impact energy had the highest values (about 150 mJ for the control and 30 mJ for the notched specimen) among the three all-ceramic specimens under the same experiment conditions. 

**Figure 6:**
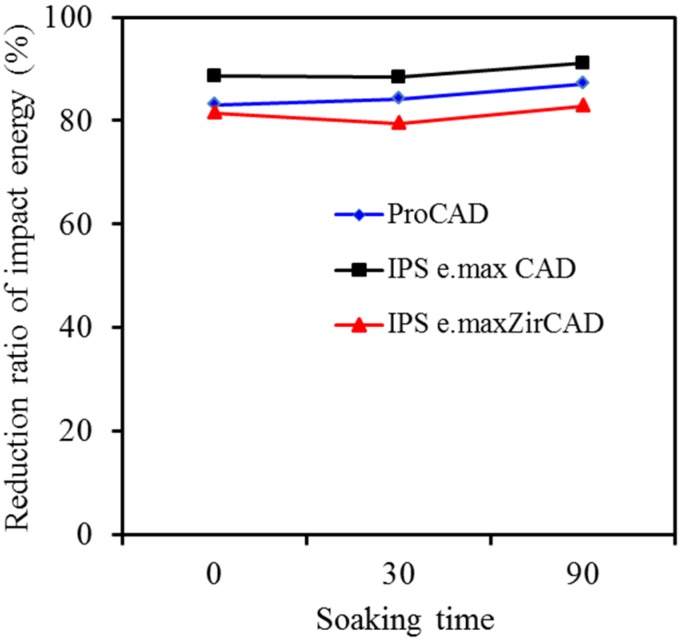
Effect of the preformed notch on reduction of impact energy of ProCAD, IPS e.max CAD and IPS e.max ZirCAD after soaking in pH 7 artificial saliva.

To further clarify the effect of the preformed notch on the impact energy of different material types after soaking, the reduction ratio of the impact energy was calculated for the all-ceramic materials with and without notch, when soaked in artificial saliva of pH 7. At a glance, it seems that the reduction ratio of impact energy of the three differently all-ceramic materials was not appreciably related to soaking time after the introduction of 0.5 mm depth notch (Figure 6), revealing a reduction in the ranging of 80%–90%.

### 3.3. pH Effect

**Figure 7:**
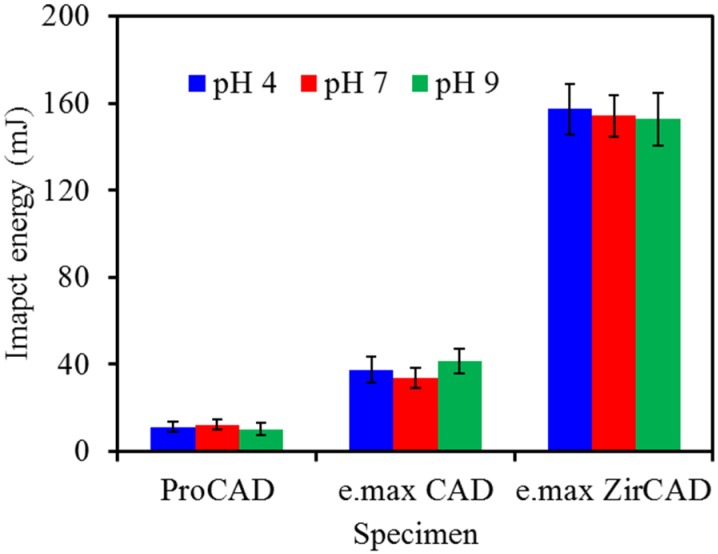
The variations in the impact energy of the three all-ceramic dental restorations without notch after soaking in artificial saliva with different pH values for 90 days.

The aim of this study was also to determine effect of artificial saliva of different pH values on the impact energy of the all-ceramic materials. Thus, the specimens without notch were soaked in artificial saliva of pH 4, 7 and 9 for 90 days. Figure 7 shows that there are no statistically significant differences (*p* > 0.05) between the test solutions with different pH values, which was independent on the used all-ceramic dental materials. 

## 4. Discussion

The use of the all-ceramic restorations has been steadily increasing in the decades. The highly esthetic ceramic glass blocks or the high-strength zirconia for dental restoration have been developed to satisfy the clinical requirements [3]. Thus, clinically relevant *in vitro* test methods are suggested to study the mechanical durability of the materials [23]. This is because that the differences in the composition, microstructure, and environmental conditions may affect the degree of degradation of the dental ceramics in an aqueous environment, particularly for the clinical long-term performance [24]. For example, low temperature degradation and mechanical and thermal cycling might decrease the strength of zirconia-based restorations, which in turn, damage their long-term success in the clinical applications [25]. Actually, ceramic corrosion/dissolution can weaken the fracture strength of these materials [26], which can affect the survival of restorations and damage to adjacent oral structures [27]. It is also speculated that the presence of the notch or flaw is one of the factors that may affect the mechanical properties of the all-ceramic materials. Therefore, this study was designed to evaluate the effect of soaking time, notch and saliva pH values on the impact energy of three commonly used all-ceramic materials for CAD/CAM.

First of all, from the results of the morphology, the leucite-reinforced ceramic (ProCAD) was more susceptible to erosion under an aqueous environment compared to the lithium-disilicate (IPS e.max CAD) and zirconia-based ceramics (IPS e.max ZirCAD). The ProCAD and IPS e.max CAD surfaces likely took place a generalized dissolution and/or ion-exchange interaction, in agreement with the earlier reports [16,27] that have indicated a substantial release of Si over time, leading to a breakdown of the glass phase. In contrast, IPS e.max ZirCAD had not obvious surface change after soaking for 90 days, possibly because the tetragonal-monoclinic phase transformation did not occur under the current soaking conditions [28]. That is that zirconia ceramics are relatively resistant to the erosion in the aqueous environment [29].

To closely mimic *in vivo* conditions and monitor material stability, the impact tests were used to investigate the mechanical properties of three different all-ceramic materials. More importantly, the impact resistance represents the total energy absorbed by a material before it fractures, when struck by a sudden biting [30]. It is the first time to systematically evaluate the impact energy of the all-ceramic materials. The present study indicated that the impact energy of all-ceramic restorations depended on the used ceramic materials and the test conditions. IPS e.max ZirCAD showed the highest impact energy, followed by IPS e.max CAD and ProCAD before and after soaking in artificial saliva. The main reason was possibly due to differences in material composition, fabrication technique and strengthening mechanism. Many studies on the investigation of flexural strength has elicited that zirconia-based ceramic are superior to the lithium disilicate-based and leucite-reinforced glass-ceramic [31,32]. It is reasonable to consider that the energy absorption during the failure process of zirconia-based ceramics was notably greater than the other two ceramic systems under impact loading. The high impact energy was particularly important when zirconia-based ceramic was used for load-bearing implant applications.

Regarding the soaking time effect, it did affect the impact energy of ProCAD. According to the literature [27,29], the leucite component of ProCAD glass ceramic dissolves easily after soaking in artificial saliva, which in turn influencing the surface structure and impact-resistant capability. In addition, the penetration of water/ions possibly accounted for the deterioration in impact energy of ProCAD. The presence of water at the tip of a crack under stress results in the rupture of the metallic oxide bonds of the dental ceramics [26]. Drummond *et al*. found that that the effect of testing in water and aging for three months caused a moderate reduction in the mean flexure strength (6%–17%) of leucite-enforced and lithium disilicate-based ceramics [14]. Conversely, the formation of the small pitting pores on the surface of IPS e.max CAD was not enough to affect its impact energy. The discrepancy in lithium disilicate-based ceramic material between this study and previous study might reside in the different materials and test environments.

According to clinical therapeutic purposes, dental porcelains are used to create replicas of natural teeth for both veneers and crown applications. In order to place the porcelain crown, at least 2 mm of tooth structure thickness is needed [33], because the thickness of the veneering porcelain and the ceramic core is related to the aesthetic appearance and mechanical properties of prosthesis. A minimum connector height of 3 to 4 mm from the interproximal papilla to the marginal ridge is suggested as the guideline for most systems [10,32,34]. Thus, a 3-mm-thick specimen was adopted in the present study to determine the impact energy of the three all-ceramic restorations. More importantly, notch sensitive property on impact energy of the materials was also investigated. Fracture initiation sites of dental ceramics are controlled primarily by the location and size of the critical flaw [35]. It is also suggested that the tendency of the material to crack or fracture at these sites of concentrated stress could be demonstrated by the relationship of results between notched and unnotched samples [21]. The present results confirmed the significant effect of one-sixth notch (0.5 mm out of 3 mm) on the reduction of the impact energy. Indeed, the impact energy values of the specimens with notch were smaller than those of the respective specimens without notch to a large extent. Surprisingly, the preformed notch led to the reduction of impact energy by 80%–90%, almost regardless of the kinds of the all-ceramic restorations. The surface of ceramic specimens with flaws could not bear excessive occlusal forces. 

The oral cavity is a potentially very hostile environment because of the moisture, coupled with change in the pH and temperature. Some reports have investigated the influence of the pH on the release of ions from various dental ceramics and slow crack growth, and there are no consistent results. The glass-phase ceramics are more prone to dissolution than the oxide ceramics and an acid environment results in more ionic release than neural environment [24,29]. On the other hand, the high pH solution is deleterious to the dissolution of glass-based systems because of breaking up the silica glass framework [27]. For the investigation of crack growth, Tomozawa *et al*. found that alkaline solutions dissolved the surface of glasses, resulting in slow crack growth and consequently reducing the material’s strength [36]. In contrast, Simmons and Freiman reported that for soda-lime glass crack velocity may not vary as function of solution pH [37]. Pinto *et al*. concluded that the effect of pH (3.5, 7.0 or 10.0) in the test environment on the stress corrosion susceptibility depended on the dental ceramics studied [26]. In the present study, it seems the saliva pH did not induce the obvious changes in the impact energy after soaking for 90 days, which indicated no statistically significant differences (*p* > 0.05) for each group. To clarify the viewpoint, further investigations, such as long-term soaking time periods and pH variations, are needed to elucidate the underlying details.

## 5. Conclusions

The chemical composition and microstructure of materials have been advocated as crucial to the clinical long-term performance of all-ceramic dental restorations. In this study, we have explored the impact energy of three all-ceramic restorations in response to soaking time, notch and saliva pHs for the first time. Within the limitations of this experimental design, soaking time exerted different effects on the impact energy of all-ceramic dental restorations. The impact energy can be affected noticeably by the preformed notch, although it also demonstrated a pH-independent result. The ability of the ceramic materials to withstand the presence of flaws propagation is an important factor affecting the clinical performance. For more understanding of the aqueous environment responsible for the decreased impact properties, further works, such as long-term soaking time periods and pH variations, will be needed.
